# Brain activation patterns in patients with post-stroke cognitive impairment during working memory task: a functional near-infrared spectroscopy study

**DOI:** 10.3389/fneur.2024.1419128

**Published:** 2024-08-12

**Authors:** Yuanyuan Liu, Zongye Zhong, Jian Chen, Hochieh Kuo, Xiuli Chen, Ping Wang, Mingfang Shi, Mingzhen Yang, Bangzhong Liu, Guanghua Liu

**Affiliations:** ^1^Department of Rehabilitation Medicine, Zhongshan Hospital, Fudan University, Shanghai, China; ^2^Department of Rehabilitation Medicine, Shanghai Geriatric Medical Center, Shanghai, China; ^3^Shanghai Institute of Rehabilitation with Integrated Western and Chinese Traditional Medicine, Shanghai, China

**Keywords:** post-stroke cognitive impairment, functional near-infrared spectroscopy, working memory, immediate recall, cognitive neuroscience

## Abstract

**Objective:**

To explore the activation patterns in the frontal cortex of patients with post-stroke cognitive impairment during the execution of working memory tasks.

**Methods:**

15 patients with post-stroke cognitive impairment, 17 patients without cognitive impairment, and 15 healthy controls of similar age and sex were included. All participants under-went immediate recall task testing and near-infrared spectroscopy imaging to measure frontal cortex activation during the task.

**Results:**

The healthy control group performed the best in the immediate recall task, followed by the post-stroke non-cognitive impairment group. The post-stroke cognitive impairment group had the poorest performance. The near-infrared spectroscopy results revealed that during the immediate recall task, the healthy control group primarily activated the left frontal lobe region. In contrast, post-stroke patients exhibited reduced activation in the left frontal lobe and increased activation in the right frontal cortex, particularly in the right frontopolar and orbitofrontal regions, with the post-stroke cognitive impairment group displaying the most pronounced changes.

**Conclusion:**

Patients with post-stroke cognitive impairment exhibit reduced activation in the left prefrontal cortex during the working memory tasks. They rely on compensatory activation in the right prefrontal cortex, particularly in the frontopolar and orbitofrontal cortex, to successfully complete the task.

## 1 Introduction

Post-stroke cognitive impairment (PSCI) refers to a series of impairments in cognitive functions, including thinking, memory, learning, attention, judgment, and language, occurring within 6 months after a stroke (1). These impairments may have a negative impact on daily life and independent functioning, severely affecting the quality of life of patients, and imposing a heavy burden on families and society (2). Studies have shown that the incidence of post-stroke cognitive impairment is around 30–50%, with dementia rates as high as 8.2% in mild stroke survivors and 34.4% in severe stroke survivors (3). PSCI involves multiple cognitive domains, such as attention, perception, memory, executive function, reasoning, language, visuospatial abilities, and so on. Among these, post-stroke memory dysfunction (PMD) is one of the most common cognitive impairments among stroke survivors (4, 5). Within the first 3 months after stroke onset, 22–55% of individuals with cognitive impairment are affected by memory dysfunction (6). PSCI not only severely affects the quality of life and social participation of patients (7), but also has a detrimental impact on the recovery of other functions, such as motor function (8).

After a stroke occurs, the presence of the damaged area leads to changes in cortical activation levels and activation patterns in patients (9). Over time, cortical function undergoes reorganization, ultimately affecting functional recovery (10). With the development of neuroimaging techniques, research on cortical reorganization after stroke has gradually become more in-depth. Among them, functional magnetic resonance imaging (fMRI) and positron emission tomography (PET) are commonly employed in researching cognitive impairment after stroke due to their high spatial resolution (11–13). However, these two techniques also have limitations. PET involves radiation exposure, while fMRI cannot be used in cases where there are intracranial metal implants. Additionally, these two techniques are costly and not suitable for long-term observations during the stroke recovery period. Lastly, both examinations require the patient to keep their body and head still, making them applicable only for real-time observation of a limited number of cognitive tasks. Functional near-infrared spectroscopy (fNIRS) is a novel non-invasive optical imaging technology that detects real-time changes in cerebral oxyhemoglobin (HbO) and deoxyhemoglobin (HbR) during brain activity. It indirectly reflects changes in cerebral oxygen consumption and hemodynamics, allowing the investigation of brain function during cognitive activities. fNIRS is affordable, portable, and less susceptible to motion artifacts, making it suitable for real-time monitoring of brain activation during movement. There have been numerous studies using fNIRS in the investigation of cognitive impairment after stroke (14–18). Most of these studies focused on resting-state brain functional connectivity in patients with PSCI (14, 17), with limited research regarding brain activation patterns during real-time working memory tasks. Even in the few studies that investigated this aspect, the regions of interest were mainly limited to the dorsolateral prefrontal cortex, while studies on other subregions of the prefrontal cortex, particularly the frontopolar cortex and orbitofrontal cortex, are scarce (16).

Therefore, in this study, we compared the performances of healthy controls, post-stroke non-cognitive impairment patients, and post-stroke cognitively impaired patients in the immediate recall task. We used fNIRS to concurrently monitor the activation patterns of different subregions in the prefrontal cortex of participants while performing the task. This research intends to explore the changes in verbal working memory abilities in PSCI patients and the alterations in cortical activation patterns during immediate recall tasks. The ultimate goal is to provide a more convenient, sensitive, and objective approach for the assessment and diagnosis of PSCI patients and to shed light on the brain functional remodeling mechanisms underlying working memory recovery in PSCI.

## 2 Materials and methods

### 2.1 Ethics statement

This study obtained ethical clearance from the Ethics Committee of Zhongshan Hospital, Fudan University. Prior to participating in the trial, all subjects were provided with information about the trial’s purpose and gave their informed consent. All participants or their family members have obtained informed consent and have signed written consent forms.

### 2.2 Participants with stroke

This study enrolled 32 patients with ischemic stroke who were admitted to the Rehabilitation Ward of Zhongshan Hospital, Fudan University between October 2022 and January 2023. All patients met the diagnostic criteria outlined in the ‘Chinese Guidelines for the Diagnosis and Treatment of Acute Ischemic Stroke 2018’. Cognitive function was assessed using the Mini-Mental State Examination (MMSE) and the Chinese version of the Montreal Cognitive Assessment Basic (MoCA-B). According to the scoring criteria, a MMSE score of ≥26 and a MoCA-B score of ≥20 (for individuals with ≤6 years of education), ≥23 (for individuals with >6 and ≤ 12 years of education), or ≥ 25 (for individuals with >12 years of education) are considered as normal cognitive function (19). Otherwise, it indicates cognitive impairment. In all, 15 patients with post-stroke cognitive impairment and 17 patients with normal cognitive after stroke were included, forming the post-stroke cognitive impairment group (PSCI group) and the post-stroke non-cognitive impairment group (Non-PSCI group), respectively.

Inclusion criteria for this study are as follows: (1) Ischemic stroke with lesions not involving bilateral prefrontal cortex; (2) Stable phase of stroke (6–12 months) and able to sit independently, capable of cooperating with scale assessments and near-infrared spectroscopy examination; (3) Willing to participate in this study; (4) Age between 40 and 80 years; and (5) Right-handed.

Exclusion criteria: (1) Hemorrhagic stroke; (2) Lesions involving the prefrontal cortex; (3) Hearing impairment or aphasia; (4) Presence of psychiatric disorders such as schizophrenia, conversion disorder, anxiety disorder, depression, etc.; (5) Patients with cognitive impairment due to other diseases such as Alzheimer’s disease, Parkinson’s disease, traumatic brain injury, etc.; and (6) Patients with skull defects.

### 2.3 Healthy participants

We concurrently recruited 15 healthy volunteers who were age and gender-matched as a healthy control group (HC group) in the community.

The inclusion criteria for the control group were as follows: (1) Cognitively normal healthy middle-aged and elderly individuals; (2) Voluntarily participate in this study; (3) Age between 40 and 80 years; and (4) Right-handed.

The exclusion criteria for the control group were as follows: (1) Presence of cognitive impairment: MMSE less than 26 points, along with MoCA-B scores less than 20 points (for individuals with ≤6 years of education), less than 23 points (for individuals with >6 years and ≤ 12 years of education), and less than 25 points (for individuals with >12 years of education); (2) History of previous stroke or traumatic brain injury; (3) Hearing impairment or aphasia; and (4) Presence of psychiatric disorders such as schizophrenia, conversion disorder, anxiety disorder, depression, etc.

### 2.4 Immediate recall task

We employed an immediate word recall task to assess the participants’ verbal working memory capacity. Previous research has suggested that the immediate word recall task involves processes of encoding, maintenance, and retrieval of words, making it an effective measure of working memory capacity. It is equally effective as other complex tasks that assess working memory span (20). The vocabulary used in the test was selected from the Auditory Verbal Learning Test – Huashan (AVLT-H) version. This set of vocabulary consists of 12 commonly used double-character words that are dissimilar in pronunciation and meaning, and is widely used in China to assess short-term and long-term memory (21). During the test, the examiner read the words clearly at a pace of 2 s per word. After reading all 12 words, the participant was immediately asked to freely recall the words. Each correctly recalled word was scored as one point, while incorrectly recalled words do not receive any points. The total time taken by the participant for the recall process was recorded as reaction times. The duration of word presentation and recall was kept within 90 s. Each participant was required to undergo a training session of at least 20 min before the formal experiment (using phrases that were not part of the test words) to ensure understanding and mastery of the entire test process.

### 2.5 fNIRS measurement

**Figure 1:**
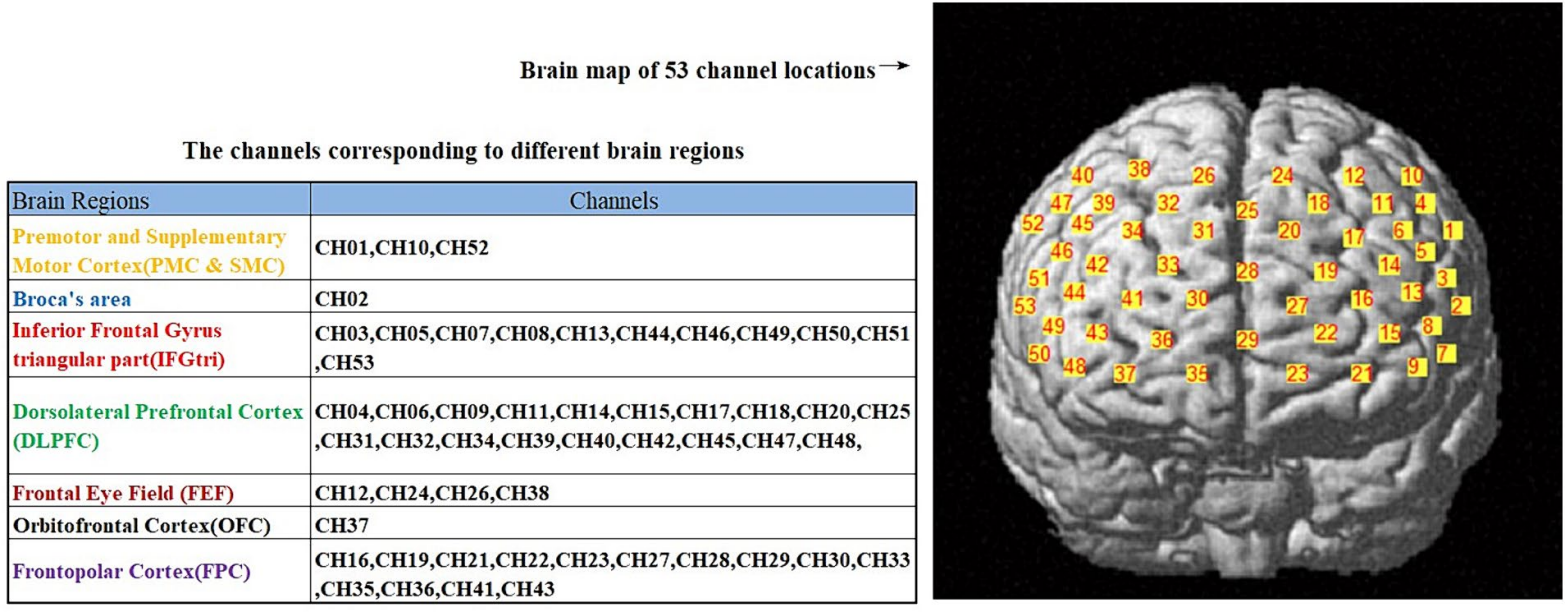
The distribution of 53 channels in the brain regions, as well as the corresponding brain functional areas for each channel.

A 53-channel NIRS system (BS-3000, Wuhan Zilian Technology Co., Ltd.) was used to evaluate frontal cortex activation during immediate recall task. The NIRS system consisted of 16 light sources and 16 detector probes. The detection wavelengths were 690 nm and 730 nm. For the positioning of fNIRS optodes, the 10/20 international system, typically utilized in electroencephalography, was utilized. The distance between adjacent light source and detector probes was 3 cm, with the lowest positioning of the probes executed along the Fp1–Fp2 line. The pathlength factor (DPF) was fixed at 6. To ensure the standardization of fNIRS channels, we employed a 3D digitizer (NirMap, Wuhan Zilian Technology Co., Ltd.) to precisely record the spatial coordinates of 4 reference points (Nz, Cz, AL, and RL) and 32 probes, which consisted of 16 light sources and 16 detector probes. Subsequently, the NIRS-SPM software was employed to transform the 53 channels into the standardized Montreal Neurological Institute (MNI) space. The regions of interest were positioned within the frontal cortical regions. The variation in oxyhemoglobin (HbO) concentrations and deoxyhemoglobin (HbR) concentrations were assessed throughout the task. As HbO was found to be a more sensitive parameter for blood flow measurement (22), we utilized the change in HbO as an indicator of frontal cortex activation. Figure 1 illustrate the channel distribution.

### 2.6 Experimental paradigm

**Figure 2:**
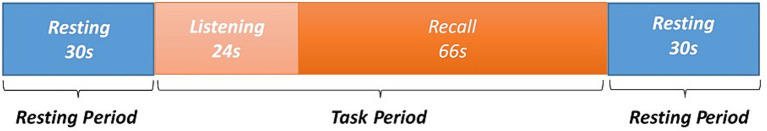
Paradigm diagram for immediate recall task.

The experimental process was divided into three parts. During the first segment of the experiment, participants were given instructions to sit in an upright position, facing forward. They were explicitly asked to keep their head, limbs, and torso still and abstain from talking or engaging in any form of distracting thinking for a period of 30 s. Then, the participant maintained the specified posture as the examiner orally presented a word every 2 seconds. Once all 12 words had been read aloud, the participant promptly commenced the recall process, which lasted for a duration of 90 s. Throughout this period, the experimenters noted the subject’s reaction time and recorded each word that was accurately recalled. Lastly, a break was implemented, during which the subject was instructed to stop the recall process and maintain an upright sitting posture. The subject was specifically advised to refrain from any form of movement, talking, or allowing distracting thoughts for a period of 30 s. Throughout the entirety of the experiment, fNIRS was employed to track variations in the HbO levels of the frontal cortex. Paradigm diagram for immediate recall task please refer to Figure 2.

### 2.7 Data processing

The analysis of the fNIRS data was carried out using the open-source software HOMER2, which is implemented in MATLAB. The first step involved converting the raw light intensity data into variations in optical density. Next step, motion artifacts were eliminated by implementing spline interpolation. To accomplish this, a bandpass filter (0.01–0.1 Hz) was utilized to remove both low-frequency and high-frequency noise resulting from movements, respiration, cardiac activity, while also reducing physiological brain spontaneous oscillations and correcting drift artifacts. In the following step, the optical density data were converted into changes in HbO concentration using the Modified Beer–Lambert Law. A generalized linear model (GLM) was then defined with the response variable being the changes in HbO concentration. Using this GLM, we analyzed the variations in HbO levels. The actual measurements of HbO concentration changes during the immediate recall task were convolved with the predicted changes to estimate neural activity response patterns under these task conditions. The least squares method was employed to estimate the response intensity (β values) parameters within the GLM framework. Finally, statistical inference was conducted on these estimated model parameters to identify brain regions showing significant changes in oxygenated hemoglobin concentration during the immediate recall task.

### 2.8 Data analysis and statistics

Statistical analysis of the data was performed using SPSS 25.0 software. The normal distribution was assessed using Shapiro–Wilk tests. A chi-square test was used to analyze the gender distribution among three groups, as well as the hemisphere distribution of infarctions in two groups of post-stroke patients. Paired sample *t*-tests were utilized to compare the cortical activation of participants in each group during the immediate recall task with the resting state. The statistical values obtained from the t-tests were visualized using the NIRS_KIT software as 2D results. The Brain Net Viewer software was employed for 3D visualization of the results. Levene’s test was used to assess the homogeneity of variance. A comparison was made among three groups of participants in terms of age, years of education, MMSE score, MoCA-B score, immediate recall score, reaction time, and channel activation during the task. One-way analysis of variance (ANOVA) was conducted, followed by post-hoc tests for pairwise comparisons among groups that showed significant differences. A Pearson correlation analysis was conducted to examine the relationship between channel activation levels and MMSE scores, MoCA-B scores, immediate recall scores, and reaction times. The level of statistical significance was set at *p* < 0.05.

## 3 Results

### 3.1 Participants

In total, 47 participants who met the inclusion criteria were included in this study. Among them, there were 17 cases with normal cognition after stroke, 15 cases experiencing cognitive impairment after stroke, and 15 cases of healthy middle-aged and elderly individuals in the control group. All stroke patients included in the study had experienced ischemic stroke. There were no significant statistical differences (*p* > 0.05) in the distribution of cerebral infarction between the two patient groups regarding the left and right hemispheres, nor in the location of infarcts, including cortical and subcortical distributions. There were no statistically significant differences (*p* > 0.05) among the three groups in terms of general characteristics, including age, gender distribution, and years of education. However, there were statistically significant differences (*p* < 0.000) in MMSE scores and MoCA-B scores among the three groups. Specifically, the scores ranked from highest to lowest as follows: HC > Non-PSCI > PSCI. Refer to Table 1 for more details.

**Table 1:** The comparison of general clinical information and immediate recall task performance between three groups.

Parameter	HC	Non-PSCI	PSCI	*F* value	*p* value
*N*	15	17	15	NA	NA
Gender(M/F)	8/7	10/7	9/6	NA	0.556
Age	64.40 ± 3.29	63.12 ± 3.89	65.07 ± 4.07	1.093	0.344
Years of Edu	13.75 ± 1.49	13.38 ± 1.69	12.85 ± 2.38	0.329	0.721
Stroke Hem(L/R)	NA	5/12	4/11	NA	0.863
Cortical/Subcortical	NA	4/13	2/13	NA	0.468
MoCA-B Scores	27.47 ± 1.52	26.43 ± 1.71^a^	23.07 ± 1.20^b^^c^	17.30	0.000^**^
MMSE Scores	28.00 ± 0.65	27.29 ± 0.99^a^	24.07 ± 0.92^b^^c^	28.10	0.000^**^
Recall Scores	4.2 ± 0.86	3.88 ± 0.78	3.07 ± 0.8^b^^c^	7.792	0.001^**^
Reaction Time(s)	23.53 ± 1.99	26.52 ± 1.62^a^	28.60 ± 1.63^b^^c^	31.62	0.000^**^

HC, healthy control; Non-PSCI, post-stroke non-cognitive impairment; PSCI, post-stroke cognitive impairment; Years of Edu, years of education; Stroke Hem, Stroke Hemisphere. MMSE, Mini-Mental State Examination; MoCA-B, Chinese version of Montreal Cognitive Assessment Basic.^**^ *p* < 0.01.

a Significant difference between Non-PSCI and HC.

b Significant difference between PSCI and HC.

c Significant difference between PSCI and Non-PSCI.

### 3.2 Immediate recall task performance

A comparative analysis was conducted on immediate recall scores and reaction times among the three groups. The results showed that in terms of immediate recall scores, the HC group had the highest scores, followed by the Non-PSCI group, and then the PSCI group. There was a statistically significant difference (*p* < 0.000) in immediate recall scores between the PSCI group and the other two groups. However, there was no statistically significant difference (*p* > 0.05) in immediate recall scores between the Non-PSCI group and the HC group. Regarding reaction times, the HC group had the shortest time, followed by the Non-PSCI group, and then the PSCI group. There was a statistically significant difference (*p* < 0.000) in reaction times among the three groups. Please refer to Table 1 for detailed results.

### 3.3 fNIRS results within groups

**Figure 3:**
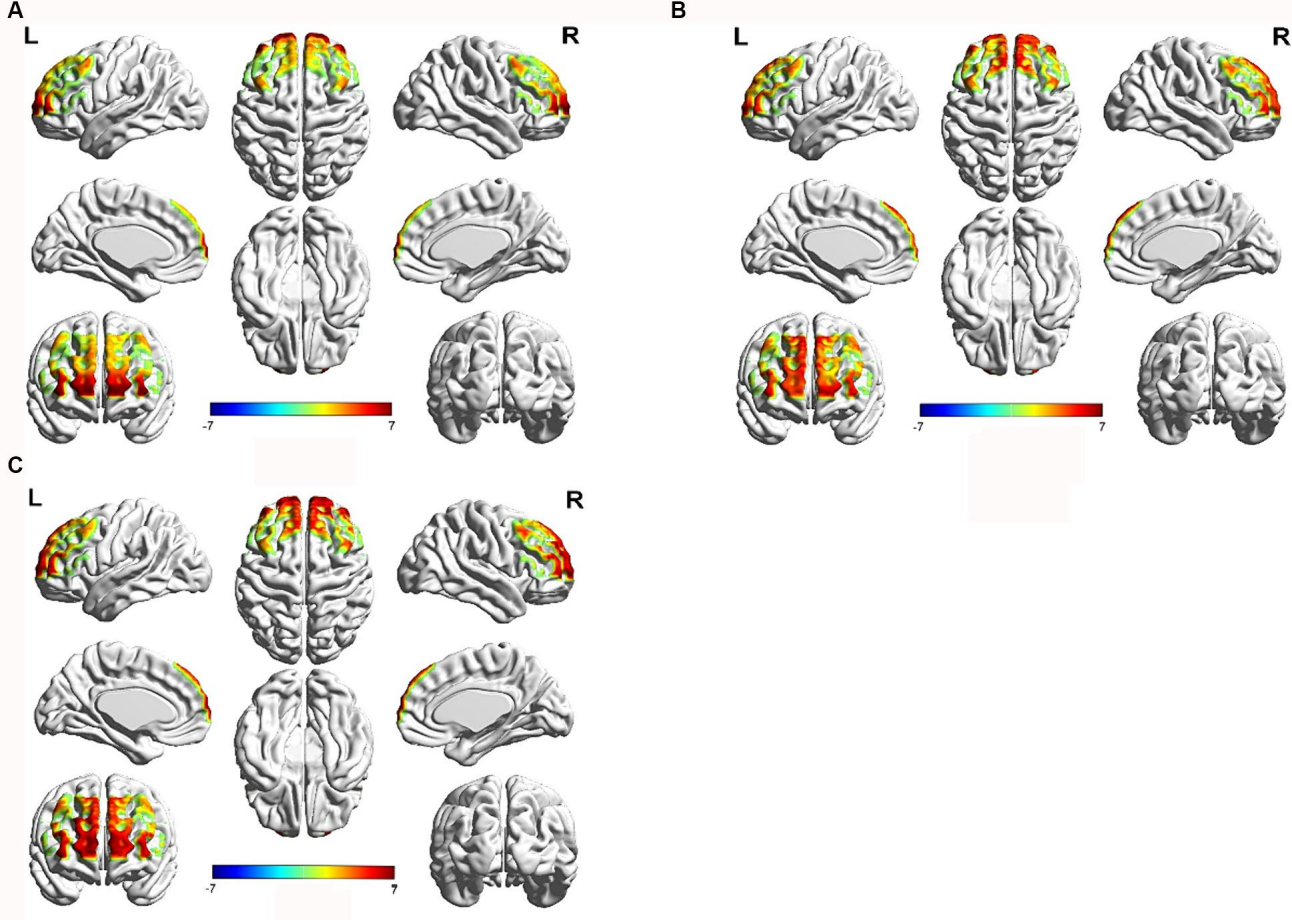
Brain activation map of the frontal cortex during immediate recall task compared to the resting state. Red color represents strong activation intensity, while blue color represents weak activation intensity. **(A)** HC group, **(B)** Non-PSCI group, **(C)** PSCI group.

Compared to the resting state, during the immediate recall task, the HC group showed significant activation (*p* < 0.05) in channels 7 and 8 (left inferior frontal gyrus triangular part), channel 9 (left dorsolateral prefrontal cortex), channels 16 and 27 (left frontopolar cortex), channel 34 (right dorsolateral prefrontal cortex), and channel 46 (right inferior frontal gyrus triangular part). The Non-PSCI group exhibited significant activation (*p* < 0.05) in channel 2 (part of the left Broca’s area), channel 13 (left inferior frontal gyrus triangular part), channel 15 (left dorsolateral prefrontal cortex), channels 16 and 21 (left frontopolar cortex), channels 30, 35, 41, and 43 (right frontopolar cortex), and channel 44 (right inferior frontal gyrus triangular part). The PSCI group showed significant activation (*p* < 0.05) in channel 2 (part of the left Broca’s area), channel 3 (left inferior frontal gyrus triangular part), channels 30, 35, 41, and 43 (right frontopolar cortex), channel 37 (right orbitofrontal cortex), and channels 44, 49, and 53 (right inferior frontal gyrus triangular part). The activation maps of the frontal lobe during the immediate recall task compared to the resting state for each group can be seen in Figure 3.

### 3.4 fNIRS results between the three groups

**Figure 4:**
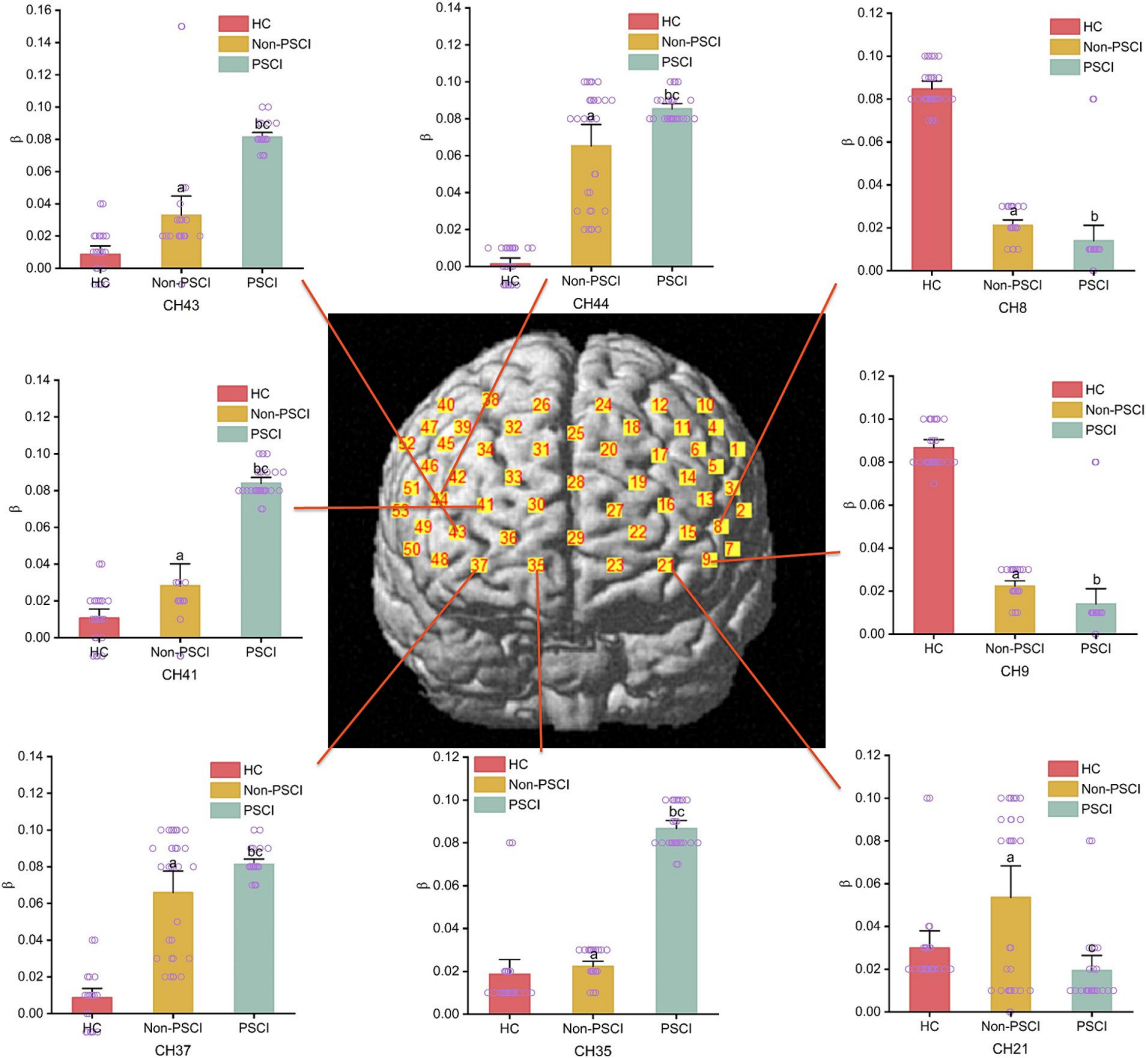
The intergroup comparison of channel activation during immediate recall task among three groups. **(a)** Inter-group comparison (HC vs. Non-PSCI); **(b)** inter-group comparison (HC vs. PSCI); **(c)** inter-group comparison (Non-PSCI vs. PSCI).

During the execution of the immediate recall task, there were group differences in the activation of specific channels among the three groups. Compared to the HC group, individuals in Non-PSCI group showed significantly enhanced activation in channel 21 (left frontopolar cortex), channels 41 and 43 (right frontopolar cortex), channel 37 (right orbitofrontal cortex), and channel 44 (right inferior frontal gyrus triangular part) (*p* < 0.01). Conversely, they exhibited significantly weakened activation in channel 8 (left inferior frontal gyrus triangular part) and channel 9 (left dorsolateral prefrontal cortex) (*p* < 0.01). Similarly, compared to the HC group, individuals in PSCI group showed significantly enhanced activation in channels 35, 41, and 43 (right frontopolar cortex), channel 37 (right orbitofrontal cortex), and channel 44 (right inferior frontal gyrus triangular part) (*p* < 0.01), while demonstrating significantly weakened activation in channel 8 (left inferior frontal gyrus triangular part) and channel 9 (left dorsolateral prefrontal cortex) (*p* < 0.01). Furthermore, compared to individuals in Non-PSCI group, patients in PSCI group exhibited significantly enhanced activation in channels 35, 41, and 43 (right frontopolar cortex), channel 37 (BA11, right orbitofrontal cortex), and channel 44 (right inferior frontal gyrus triangular part) (*p* < 0.01), while showing significantly weakened activation in channel 21 (left frontopolar cortex) (*p* < 0.01). Please refer to Table 2 and Figure 4 for more details.

**Table 2:** Channels were significantly activated in the task state between three groups.

CH	HC	Non-PSCI	PSCI	F	*p*
CH8	0.084 ± 0.009	0.019 ± 0.006^a^	0.013 ± 0.018^b^	35.78	0.000**
CH9	0.086 ± 0.009	0.021 ± 0.006^a^	0.013 ± 0.018^b^	28.16	0.001**
CH21	0.026 ± 0.006	0.079 ± 0.028^a^	0.017 ± 0.016^c^	46.57	0.000**
CH35	0.017 ± 0.015	0.022 ± 0.006	0.087 ± 0.008^b^^c^	76.22	0.000**
CH41	0.008 ± 0.013	0.033 ± 0.021^a^	0.081 ± 0.007^b^^c^	44.36	0.000**
CH43	0.008 ± 0.013	0.065 ± 0.032^a^	0.080 ± 0.007^b^^c^	48.87	0.013*
CH37	0.010 ± 0.012	0.027 ± 0.032^a^	0.083 ± 0.007^b^^c^	46.77	0.000**
CH44	0.007 ± 0.015	0.066 ± 0.032^a^	0.082 ± 0.006^b^^c^	75.59	0.000**

HC, healthy control; Non-PSCI, post-stroke non-cognitive impairment; PSCI, post-stroke cognitive impairment; CH, channel.^*^ *p* < 0.05, ^**^ *p* < 0.01.

a Significant difference between Non-PSCI and HC.

b Significant difference between PSCI and HC.

c Significant difference between PSCI and Non-PSCI.

### 3.5 Results of correlation analysis

A correlation analysis was conducted to investigate the relationship between MMSE, MoCA-B scores, immediate recall scores, reaction times, and channel activation. The results showed that MMSE and MoCA-B scores were positively correlated with the activation in the left inferior frontal gyrus triangular part (IFGtri-L), left dorsolateral prefrontal cortex (DLPFC-L), and left frontopolar cortex (FPC-L), while negatively correlated with the activation in the right frontopolar cortex (FPC-R), right orbitofrontal cortex (OFC-R), and right inferior frontal gyrus triangular part (IFGtri-R). Immediate recall scores were positively correlated with the activation in the left inferior frontal gyrus triangular part (IFGtri-L) and left dorsolateral prefrontal cortex (DLPFC-L), but negatively correlated with the activation in the right orbitofrontal cortex (OFC-R) and right inferior frontal gyrus triangular part (IFGtri-R). On the other hand, reaction times showed an inverse correlation with the above measures compared to the correlation between immediate recall scores and these measures. These findings are summarized in Table 3.

**Table 3:** Correlation analysis between MMSE scores, MoCA-B scores, immediate recall task performance and channel activation.

Parameter	IFGtri-L (CH8)	DLPFC-L (CH9)	FPC-L (CH21)	FPC-R (CH35)	OFC-R (CH37)	IFGtri-R (CH44)
MMSE	0.606**	0.625**	0.338*	−0.842**	−0.787**	−0.574**
MoCA-B	0.625**	0.634**	0.407**	−0.853**	−0.782**	−0.642**
Recall Scores	0.406**	0.410**	0.136	−0.263	−0.284*	−0.385*
Reaction Time	−0.638*	−0.654**	−0.151	0.536**	0.530**	0.533**

IFGtri-L/R, left/right inferior frontal gyrus triangular part; DLPFC-L, left dorsolateral prefrontal cortex; FPC-L/R, left/right frontopolar cortex; OFC-R, right orbitofrontal cortex; MMSE, Mini-Mental State Examination; MoCA-B, Chinese version of Montreal Cognitive Assessment Basic; ^*^ *p* < 0.05, ^**^ *p* < 0.01.

## 4 Discussion

After a stroke, damage to specific brain regions can impair working memory capacity and alter the brain activation pattern during the working memory process (17). In a study by Ying et al., they administered working memory tasks such as the clock drawing test and digit span test to patients with PSCI. They found that compared to healthy individuals, PSCI patients exhibited significant activation in the right frontal lobe, particularly in the sensorimotor area and the prefrontal cortex. This suggests that PSCI patients engage additional brain regions to compensate for the decline in cognitive abilities (17). Li et al. discovered that PSCI patients with involvement of the right hemisphere exhibited greater activation in the prefrontal lobe, as well as bilateral activation, during the performance of the continuous performance test compared to healthy individuals (23). Functional magnetic resonance imaging studies in traumatic brain injury patients indicated that, compared to healthy controls, the brain activation patterns of traumatic brain injury patients during the paced auditory serial addition task were more dispersed and biased towards the right hemisphere, with the right frontal lobe activation being particularly prominent (24). Chen et al. found that during the 2-back task, healthy young individuals exhibited activation primarily in the left hemisphere, specifically in the frontal and parietal lobes. However, young individuals with mild traumatic brain injury (TBI) showed bilateral activation in the frontal and parietal lobes during the early stages of TBI. After a period of 6 months, when performing the same task, the young TBI patients gradually exhibited a similar activation pattern to that of healthy young individuals, primarily activating the left hemisphere (25). Our study revealed that both Non-PSCI and PSCI patients showed a gradual decline in immediate recall scores and an increase in reaction times compared to the healthy control group. This suggests a decrease in verbal working memory and a reduction in information processing efficiency in patients after stroke, with the most noticeable impairments seen in the PSCI group. Near-infrared spectroscopy imaging demonstrated that during the immediate recall task, the healthy control group primarily activated the left inferior frontal gyrus triangular part (IFGtri-L), left dorsolateral prefrontal cortex (DLPFC-L), and left frontopolar cortex (FPC-L), with minimal activation observed in the right dorsolateral prefrontal cortex (DLPFC-R) and right inferior frontal gyrus triangular part (IFGtri-R), indicating a clear left hemisphere dominance. In contrast, stroke patients showed attenuated activation in the left IFGtri and left DLPFC during the task compared to the control group, while exhibiting significantly increased activation in the right hemisphere, including the right IFGtri and right frontopolar cortex (FPC-R), demonstrating prominent bilateral activation. These findings are consistent with previous research results (17, 23–25).

In PSCI patients, the brain activation patterns during working memory tasks shift from a left hemisphere lateralization seen in healthy individuals to bilateral activation or right hemisphere lateralization. This lateralized activation is particularly evident in the right frontal cortex, although the underlying reasons for this shift are still unknown. Possible explanations could be that PSCI patients experience a decrease in efficiency within the brain regions originally responsible for the task, requiring compensatory activation from other brain areas. It could also be due to differences in how PSCI patients and healthy individuals approach the same task, or a combination of both factors. Similar phenomena have been observed in studies on age-related decline in working memory. Reuter et al. found that, unlike young individuals, older adults exhibit involvement of the bilateral prefrontal cortex during tasks involving verbal working memory and long-term memory (26). There are many similar studies with similar conclusions (27–30). The currently acknowledged explanation for these findings is the Compensation Related Utilization of Neural Circuits Hypothesis (CRUNCH). According to the CRUNCH theory, age-related deterioration in brain structure and function leads to reduced neural efficiency. Therefore, when older adults perform cognitive tasks that impose similar demands as those on younger individuals, they require the recruitment of additional neural resources, resulting in greater brain activation across multiple regions (31). Perhaps this theory could also be applied to explain the changes in brain activation patterns during working memory tasks in PSCI patients.

The model of verbal working memory consists of at least three distinct components: the rehearsal component, attentional component, and processing or executive component. The rehearsal component is typically associated with Broca’s area and supplemental motor areas. It is responsible for maintaining and cyclically repeating information to prevent it from being lost in working memory. It encompasses two sub-components: phonological storage and phonological rehearsal. The former temporarily stores auditory speech information, while the latter reproduces the information during the thinking process. The attentional component is located in the posterior parietal area and is responsible for the allocation and control of attentional resources. It helps in focusing attention on relevant information and filtering out distractions during the working memory task. The executive component is located in the prefrontal area and serves as the executive center of verbal working memory. It is responsible for processing and manipulating information, making decisions, and problem-solving within the working memory context (32, 33). Our study revealed that during the immediate recall task, all three groups of participants exhibited activation in the bilateral inferior frontal gyrus triangular part, including Broca’s Area, highlighting its crucial role in verbal working memory tasks. This finding is consistent with previous research (34). Our study also showed that compared to the healthy control group, stroke patients exhibited attenuated activation in the left inferior frontal gyrus triangular part while demonstrating more pronounced activation in the right inferior frontal gyrus triangular part. Among the stroke patients, those with cognitive impairment patients showed the strongest activation in this region. Previous research has found that individuals with aphasia exhibit increased activation in the right hemisphere language homologous areas during language testing (35, 36). A systematic review by Wilson et al. revealed that the left hemisphere language areas in individuals with aphasia are less active compared to normal control groups, and there is moderate evidence suggesting that individuals with aphasia recruit right hemisphere homologous regions to varying degrees (37). Patients with cognitive impairment following subarachnoid hemorrhage show increased activation in bilateral frontal cortical areas, including the inferior frontal gyrus, during the execution of the Sternberg verbal working memory task (38). This increased activation may also serve as compensation for the inadequate activation in the corresponding left hemisphere language areas.

Our study also found that, during the performance of the same immediate recall task, stroke patients with normal cognition exhibited additional activation in the right frontopolar cortex compared to healthy controls. On the other hand, stroke patients with cognitive impairment exhibited additional activation in the right frontopolar cortex and right orbitofrontal cortex, both of which are subregions of the prefrontal cortex. The prefrontal cortex serves as the central executive system of working memory, and its functioning can be divided into two functional aspects: motivational behavior, responsible for evaluation and decision-making; and control behavior, responsible for cognitive control (39). The control behavior is associated with the dorsolateral prefrontal cortex (DLPFC) and the lateral parietal cortex (40). Motivational behavior refers to the cognitive processes related to evaluation, motivation, reward learning, and decision-making, and it is primarily regulated by the frontopolar cortex, orbitofrontal cortex, and ventromedial prefrontal cortex (41). The frontopolar cortex (FPC) is responsible for higher-order cognitive control (42), and it assists in complementing the functions of the dorsolateral prefrontal cortex in working memory tasks, including self-evaluation based on external input (metacognition) (43). Specifically, the FPC is involved in determining when to transform “external” information, such as verbal stimuli, into “internal” information, such as the maintenance of episodic memories, and it plays a role in monitoring, integrating, and decision-making processes (42). Additionally, the frontopolar region is also associated with the updating function in the central executive processes of working memory. As early as 1994, Grasby et al. found that the activity in the frontopolar cortex increased with the number of times participants recalled individual words (44). Van et al., using a serial recall procedure to test the updating function of working memory, employed PET to detect changes in cortical activation. The results revealed that the left frontopolar cortex exhibited the most significant activation during the task, with activation spreading to the left medial frontal gyrus and right frontopolar cortex. Based on these findings, it was suggested that the updating of information in working memory is mainly associated with the activity of the frontopolar cortex (45). The orbitofrontal cortex is typically associated with emotion, motivation, reward, and punishment (46). It is also closely related to higher cognitive functions, including attention, working memory, decision-making, problem-solving, and executive planning. It contributes to inhibiting impulses, regulating attention and focus, and assisting in goal-directed behaviors (47). In individuals with PSCI, there is insufficient activation of the left dorsolateral prefrontal cortex and other brain regions during immediate recall tasks. As a compensatory mechanism, there is increased activation in the right frontopolar cortex and the orbitofrontal cortex, highlights the crucial role of these two regions in verbal working memory and as a compensatory mechanism for the impaired functioning of the dorsolateral prefrontal cortex. The compensatory activation of the right frontopolar cortex and orbitofrontal cortex in PSCI patients during verbal working memory tasks, to the best of our knowledge, has not been reported in previous literature and requires further research for confirmation.

The correlation analysis results showed that the MMSE and MoCA-B scores were positively correlated with the activation in the left inferior frontal gyrus triangular part (IFGtri-L), left dorsolateral prefrontal cortex (DLPFC-L), and left frontopolar cortex (FPC-L). On the other hand, they were negatively correlated with the activation in the right frontopolar cortex (FPC-R), orbitofrontal cortex (OFC-R), and right inferior frontal gyrus triangular part (IFGtri-R). These findings indicate that cognitive performance is positively associated with a pattern of lateralization of activation in the left hemisphere, and negatively associated with bilateral or right hemisphere lateralization of activation, which is consistent with previous research (13). This further confirms that individuals with good cognitive function and good verbal working memory only require activation in the left hemisphere to complete the task, whereas those with poorer cognitive function need to recruit the right hemisphere for compensation. The correlation results between immediate recall performance and brain activation were similar to the correlation results between MMSE, MoCA-B scores, and brain activation. This suggests that the immediate recall test can be utilized to some extent in evaluating brain function in patients with PSCI.

This study has several limitations. Firstly, this study examined patients with cognitive impairments following different locations of cerebral infarction as a whole, which may not be homogeneous in reality. Secondly, our channels were primarily focused on the frontal lobe cortex, which means that we could not fully assess the activation in other brain regions or understand the interactions between different brain regions. Lastly, our sample size was relatively small, and future studies should consider expanding the sample size to further validate our results.

## 5 Conclusion

Our results indicate that patients with post-stroke cognitive impairment exhibit insufficient activation in the left prefrontal cortex during the execution of an immediate recall task. They rely on compensatory activation in the right prefrontal cortex, particularly in the frontopolar and orbitofrontal cortex, to successfully complete the task.

## Data availability statement

The raw data supporting the conclusions of this article will be made available by the authors, without undue reservation.

## Ethics statement

The studies involving humans were approved by Ethics Committee of Zhongshan Hospital, Fudan University. The studies were conducted in accordance with the local legislation and institutional requirements. The participants provided their written informed consent to participate in this study. Written informed consent was obtained from the individual(s) for the publication of any potentially identifiable images or data included in this article.

## Author contributions

YL: Writing – original draft, Visualization, Formal analysis, Conceptualization. ZZ: Writing – original draft, Visualization, Formal analysis, Conceptualization. JC: Writing – review & editing, Software, Conceptualization. HK: Writing – review & editing, Investigation, Data curation, Conceptualization. XC: Writing – review & editing, Investigation, Data curation, Conceptualization. PW: Writing – review & editing, Supervision, Resources, Conceptualization. MS: Writing – review & editing, Supervision, Conceptualization. MY: Writing – review & editing, Supervision, Conceptualization. BL: Writing – review & editing, Software, Data curation, Conceptualization. GL: Writing – review & editing, Resources, Project administration, Conceptualization.
