# Supplementary material for: Brain activation patterns in patients with post-stroke cognitive impairment during working memory task: a functional near-infrared spectroscopy study
Source: Front Neurol. 2024 Aug 12;15:1419128. doi: 10.3389/fneur.2024.1419128 (PMC11346344; doi:10.3389/fneur.2024.1419128)
Supplement: Supplementary file 1 [file Table_1.DOCX]

**Supplementary Material**

**Supplementary Table 1.** General characteristics of participants and activated channels

| **Subject** | **Group** | **Gender** | **Age** | **Disease**  **Course** | **Stroke Location** | **Activated**  **Channels** |
| --- | --- | --- | --- | --- | --- | --- |
| 1 | Non-PSCI | Male | 59 | 6 months | Periventricular (left) | CH2,CH15,  CH41,CH43 |
| 2 | Non-PSCI | Male | 60 | 6 months | Periventricular (left) | CH15,CH21,  CH43 |
| 3 | Non-PSCI | Male | 65 | 8 months | Brainstem (left) | CH21,CH30,  CH35,CH43 |
| 4 | Non-PSCI | Female | 67 | 7 months | Left frontal, parietal, and temporal lobes without the prefrontal cortex | CH2,CH35,  CH41,CH43 |
| 5 | Non-PSCI | Male | 71 | 10 months | Periventricular (left) | CH2,CH15,  CH21,CH30,  CH41,CH43 |
| 6 | Non-PSCI | Male | 66 | 8 months | Periventricular (right) | CH16,CH35,  CH41,CH43 |
| 7 | Non-PSCI | Male | 66 | 7 months | Brainstem (right) | CH2,CH21,  CH41,CH43 |
| 8 | Non-PSCI | Male | 65 | 6 months | Right frontal, parietal, and temporal lobes without the prefrontal cortex | CH2,CH13,  CH21,CH43 |
| 9 | Non-PSCI | Female | 67 | 7 months | Periventricular (right) | CH2,CH21,  CH35,CH41,  CH44 |
| 10 | Non-PSCI | Female | 63 | 8 months | Thalamus (right) | CH30,CH35,  CH43 |
| 11 | Non-PSCI | Female | 64 | 9 months | Brainstem (right) | CH30,CH35,  CH41,CH44 |
| 12 | Non-PSCI | Male | 65 | 9 months | Right frontal lobe without the prefrontal cortex | CH15,CH21,  CH35,CH43 |
| 13 | Non-PSCI | Female | 59 | 8 months | Periventricular (right) | CH21,CH35,  CH43 |
| 14 | Non-PSCI | Male | 58 | 7 months | Periventricular (right) | CH16,CH21,  CH35,CH43 |
| 15 | Non-PSCI | Female | 57 | 7 months | Right frontal, parietal, and temporal lobes without the prefrontal cortex | CH2,CH13,  CH21 |
| 16 | Non-PSCI | Male | 60 | 8 months | Periventricular (right) | CH21,CH35,  CH43,CH44 |
| 17 | Non-PSCI | Female | 61 | 7 months | Periventricular (right) | CH21,CH30,  CH43,CH44 |
| 18 | PSCI | Male | 62 | 8 months | Periventricular (right) | CH2,CH3,  CH35,CH37,  CH41,CH43,  CH44 |
| 19 | PSCI | Male | 63 | 7 months | Right frontal, parietal, and temporal lobes without the prefrontal cortex | CH3,CH44,  CH49,CH53 |
| 20 | PSCI | Male | 67 | 8 months | Periventricular (left) | CH2,CH35,  CH37,CH41,  CH43,CH44,  CH53 |
| 21 | PSCI | Male | 58 | 6 months | Brainstem (left) | CH2,CH35,  CH37,CH41,  CH44,CH53 |
| 22 | PSCI | Female | 59 | 10 months | Left frontal, parietal, and temporal lobes without the prefrontal cortex | CH30,CH35,  CH37,CH41,  CH44,CH53 |
| 23 | PSCI | Male | 62 | 8 months | Periventricular (left) | CH3,CH30,  CH35,CH37,  CH41,CH44,  CH53 |
| 24 | PSCI | Male | 67 | 9 months | Periventricular (right), Brainstem (right) | CH2,CH3,  CH30,CH35,  CH37,CH41,  CH44,CH53 |
| 25 | PSCI | Male | 71 | 8 months | Periventricular (right) | CH3,CH30,  CH35,CH37,  CH41,CH44,  CH49,CH53 |
| 26 | PSCI | Male | 72 | 7 months | Brainstem (right) | CH3,CH35,  CH37,CH41,  CH44,CH49,  CH53 |
| 27 | PSCI | Male | 65 | 8 months | Periventricular (right), Temporal lobe (right) | CH3,CH35,  CH41,CH44 |
| 28 | PSCI | Female | 66 | 6 months | Periventricular (right) | CH3,CH35,  CH41,CH44,  CH49 |
| 29 | PSCI | Female | 67 | 7 months | Periventricular (right) | CH3,CH35,  CH37,CH41,  CH44,CH49,  CH53 |
| 30 | PSCI | Female | 68 | 8 months | Brainstem (right) | CH3,CH35,  CH37,CH41,  CH44,CH49,  CH53 |
| 31 | PSCI | Female | 64 | 9 months | Periventricular (right) | CH2,CH35,  CH41,CH44,  CH49 |
| 32 | PSCI | Female | 65 | 7 months | Brainstem (right) | CH3,CH35,  CH37,CH41,  CH43,CH44,  CH49,CH53 |
| 33 | HC | Male | 67 | / | / | CH8,CH9,  CH34 |
| 34 | HC | Male | 61 | / | / | CH7,CH8,  CH9,CH46 |
| 35 | HC | Male | 60 | / | / | CH8,CH9,  CH34 |
| 36 | HC | Male | 61 | / | / | CH9,CH16,  CH34 |
| 37 | HC | Male | 62 | / | / | CH7,CH8,  CH9,CH27,  CH46 |
| 38 | HC | Male | 65 | / | / | CH9,CH16,  CH27,CH34 |
| 39 | HC | Male | 67 | / | / | CH8,CH9,  CH16,CH34 |
| 40 | HC | Male | 70 | / | / | CH9,CH16,  CH34 |
| 41 | HC | Female | 58 | / | / | CH7,CH8,  CH9,CH46 |
| 42 | HC | Female | 65 | / | / | CH9,CH16,  CH27,CH34 |
| 43 | HC | Female | 67 | / | / | CH7,CH8,  CH9,CH46 |
| 44 | HC | Female | 65 | / | / | CH9,CH34,  CH46 |
| 45 | HC | Female | 65 | / | / | CH9,CH16,  CH27,CH34 |
| 46 | HC | Female | 67 | / | / | CH7,CH8,  CH9,CH46 |
| 47 | HC | Female | 66 | / | / | CH8,CH9,  CH16,CH34 |

Abbreviation：CH, Channel; Non-PSCI, post-stroke non-cognitive impairment；PSCI, post-stroke cognitive impairment; HC，healthy control.
